# After-War Crime

**Published:** 1920-01-31

**Authors:** 


THE PUBLIC WELL-BEING—(*continued*)

### AFTER-WAR CRIME.

Various explanations have been offered regarding the serious wave of crime. Judges, social workers, and politicians have all endeavoured to trace it to its roots. It seems generally to be agreed that war inevitably tends to a laxity of morals, and it seems natural, after a war like the last, when life was so appallingly sacrificed, that men should now have less respect for the sanctity of life. To a man of low mental calibre, physical violence in civil life may appear to be a small thing by comparison.

Judge Atherley Jones attributes the growth of crimes of violence to sensationalism in the Press and on the film, which lead people of weak intellect to emulate the examples luridly reported. This may be so, but it is doubtful whether it is responsible for very much. There appears to be, unfortunately, only too clear an evidence of purpose in the majority of offences. But whatever the causes, there can be no two opinions about the Judge's view that the courts must act with severity. Mr. Justice Darling, also, has spoken wisely on the need not only of drastic punishment of loathsome and debauched individuals who indulge in nefarious practices, but of the stiffening of the law in relation to questionable advertisements. The judges are showing a healthy scepticism towards the well-worn excuse of offenders that they suffer from shell-shock, or that the war has shattered their nerves. Irritable people must be taught that such excuses will not lightly protect them, but will be subjected to the strictest test. The real fact seems to be that an accustomed use of weapons, the commonplace sight of death, and the freedom from discipline has cheapened life in the minds of men who in any event would have been potential criminals. Rigorous punishment is the solution.

